# 150. Low Propensity for Development of Spontaneous *In Vitro* Resistance to Tebipenem, Ertapenem and Meropenem Among Enterobacterales Uropathogens

**DOI:** 10.1093/ofid/ofac492.228

**Published:** 2022-12-15

**Authors:** Brian D VanScoy, Haley Conde, Rodrigo E Mendes, Nicole Cotroneo, Ian A Critchley, Ian A Critchley

**Affiliations:** Institute for Clinical Pharmacodynamics, Schenectady, New York; Institute for Clinical Pharmacodynamics, Schenectady, New York; JMI Laboratories, North Liberty, Iowa; Spero Therapeutics, Cambridge, Massachusetts; Spero Therapeutics, Cambridge, Massachusetts; Spero Therapeutics, Cambridge, Massachusetts

## Abstract

**Background:**

Tebipenem (TBP), an oral carbapenem in development for treatment of complicated urinary tract infections and acute pyelonephritis, is active vs. uropathogens which produce extended-spectrum beta-lactamases (ESBLs) such as *Escherichia coli* (Ec) and *Klebsiella pneumoniae* (Kp). Bacteria acquire resistance to antibiotics via multiple routes, including by chromosomal mutation. In this study, spontaneous resistance to TBP among Enterobacterales, including those resistant to clinically relevant antibacterials, was assessed

**Methods:**

The frequency of resistance (FOR) to TBP, meropenem (MEM) and ertapenem (ETP) was assessed for Ec (n = 8), Kp (n = 9), and other Enterobacterales (n = 7). Approximately 10^8^ CFU were plated on agar containing 4x or 8x the MIC and incubated for 24h. Resistance was confirmed by MIC testing (CLSI M07Ed11). Serial passages were performed with two Ec and two Kp isolates, each of which included a wild type and ESBL+ isolate, using TBP, MEM and ETP over a 14-day period. Whole genome sequencing was conducted and assembled mutant sequences were aligned vs. β-lactamase-encoding genes, porins OmpF/OmpK35, OmpC/OmpK36, and OmpK37 (Kp only) and penicillin binding proteins (PBPs) 1a, 1b, 2, and 3 and compared with parent strains.

**Results:**

FOR values for TBP, MEM and ETP at 4x to 8x the MIC ranged from < 6.87 × 10^-9^ to 5.54 × 10^-5^, with similar results obtained for all species and genotypes, including ESBL+ isolates and those with porin alterations. In the serial passage, MICs for each agent vs. four isolates over the 14-day period were ∼ five to seven 2x dilutions higher than baseline, resulting in similar MIC values to those observed in the FOR studies (0.12 to 2 µg/mL). Mutants isolated in the presence of TBP, MEM or ETP had similarly decreased susceptibility to each compound. In general, mutants selected during the single- and multi-step resistance studies showed alterations of OmpC in Ec and its homologue OmpK36 in Kp.

**Conclusion:**

These data suggest TBP has a low propensity for spontaneous resistance at 4x to 8x MIC *in vitro* and was similar to MEM and ETP in both single-step and serial passage studies. Cross-resistance to TBP, MEM or ETP among mutants suggests common mechanisms contribute to elevated MICs, including through reduced permeability.

**Disclosures:**

**Brian D. VanScoy, B.S.**, Adagio Therapeutics, Inc.: Grant/Research Support|Amplyx Pharmaceuticals, Inc.: Grant/Research Support|AN2 Therapeutics,: Grant/Research Support|Antabio SAS: Grant/Research Support|Arcutis Biotherapeutics, Inc.: Grant/Research Support|B. Braun Medical Inc.: Grant/Research Support|Basilea Pharmaceutica: Grant/Research Support|Boston Pharmaceuticals: Grant/Research Support|Celdara Medical LLC: Grant/Research Support|Cidara Therapeutics Inc.: Grant/Research Support|Cipla USA: Grant/Research Support|Crestone Inc.: Grant/Research Support|CXC: Grant/Research Support|Debiopharm International SA: Grant/Research Support|Entasis Therapeutics: Grant/Research Support|Evopoint Biosciences Co.: Grant/Research Support|Fedora Pharmaceuticals: Grant/Research Support|GlaxoSmithKline: Grant/Research Support|Hoffmann-La Roche: Grant/Research Support|Insmed Inc.: Grant/Research Support|Iterum Therapeutics Limited: Grant/Research Support|Kaizen Bioscience, Co.: Grant/Research Support|KBP Biosciences USA: Grant/Research Support|Lassen Therapeutics: Grant/Research Support|Matinas Biopharma: Grant/Research Support|Meiji Seika Pharma Co., Ltd.: Grant/Research Support|Melinta Therapeutics: Grant/Research Support|Menarini Ricerche S.p.A: Grant/Research Support|Mutabilis: Grant/Research Support|Nabriva Therapeutics AG: Grant/Research Support|Novartis Pharmaceuticals Corp.: Grant/Research Support|Paratek Pharmaceuticals, Inc.: Grant/Research Support|PureTech Health: Grant/Research Support|Sfunga Therapeutics: Grant/Research Support|Spero Therapeutics: Grant/Research Support|Suzhou Sinovent Pharmaceuticals Co.: Grant/Research Support|TauRx Therapeutics: Grant/Research Support|Tetraphase Pharmaceuticals: Grant/Research Support|tranScrip Partners: Grant/Research Support|Utility Therapeutics: Grant/Research Support|Valanbio Therapeutics, Inc.: Grant/Research Support|VenatoRx: Grant/Research Support|Wockhardt Bio AG: Grant/Research Support **Haley Conde, B.S.**, Adagio Therapeutics, Inc.: Grant/Research Support|Amplyx Pharmaceuticals, Inc: Grant/Research Support|AN2 Therapeutics,: Grant/Research Support|Antabio SAS,: Grant/Research Support|Arcutis Biotherapeutics, Inc.: Grant/Research Support|B. Braun Medical Inc.: Grant/Research Support|Basilea Pharmaceutica,: Grant/Research Support|Boston Pharmaceuticals: Grant/Research Support|Celdara Medical LLC: Grant/Research Support|Cidara Therapeutics Inc: Grant/Research Support|Cipla USA: Grant/Research Support|Crestone Inc.: Grant/Research Support|CXC,: Grant/Research Support|Debiopharm International SA: Grant/Research Support|Entasis Therapeutics: Grant/Research Support|Evopoint Biosciences Co.: Grant/Research Support|Fedora Pharmaceuticals: Grant/Research Support|GlaxoSmithKline: Grant/Research Support|Hoffmann-La Roche: Grant/Research Support|Insmed Inc.: Grant/Research Support|Iterum Therapeutics Limited,: Grant/Research Support|Kaizen Bioscience, Co.: Grant/Research Support|KBP Biosciences USA: Grant/Research Support|Lassen Therapeutics: Grant/Research Support|Matinas Biopharma,: Grant/Research Support|Meiji Seika Pharma Co., Ltd.: Grant/Research Support|Melinta Therapeutics: Grant/Research Support|Menarini Ricerche S.p.A: Grant/Research Support|Mutabilis: Grant/Research Support|Nabriva Therapeutics AG: Grant/Research Support|Novartis Pharmaceuticals Corp.: Grant/Research Support|Paratek Pharmaceuticals, Inc.: Grant/Research Support|PureTech Health: Grant/Research Support|Sfunga Therapeutics: Grant/Research Support|Spero Therapeutics: Grant/Research Support|Suzhou Sinovent Pharmaceuticals Co.: Grant/Research Support|TauRx Therapeutics: Grant/Research Support|Tetraphase Pharmaceutical: Grant/Research Support|tranScrip Partners: Grant/Research Support|Utility Therapeutics: Grant/Research Support|Valanbio Therapeutics, Inc.: Grant/Research Support|VenatoRx: Grant/Research Support|Wockhardt Bio AG: Grant/Research Support **Rodrigo E. Mendes, PhD**, Spero Therapeutics: Grant/Research Support **Nicole Cotroneo, B.S.**, Spero Therapeutics: Employee|Spero Therapeutics: Stocks/Bonds **Ian A. Critchley, PhD**, Spero Therapeutics: Employee|Spero Therapeutics: Stocks/Bonds **Ian A. Critchley, PhD**, Spero Therapeutics: Employee|Spero Therapeutics: Stocks/Bonds.

